# Contemporary Trends in Global Mortality of Sepsis Among Young Infants Less Than 90 Days: A Systematic Review and Meta-Analysis

**DOI:** 10.3389/fped.2022.890767

**Published:** 2022-06-03

**Authors:** Ming Ying Gan, Wen Li Lee, Bei Jun Yap, Shu Ting Tammie Seethor, Rachel G. Greenberg, Jen Heng Pek, Bobby Tan, Christoph Paul Vincent Hornik, Jan Hau Lee, Shu-Ling Chong

**Affiliations:** ^1^Yong Loo Lin School of Medicine, National University of Singapore, Singapore, Singapore; ^2^Duke-NUS Medical School, Singapore, Singapore; ^3^Department of Paediatrics, Duke University School of Medicine, Durham, NC, United States; ^4^Emergency Medicine, Sengkang General Hospital, Singapore, Singapore; ^5^Department of Paediatrics, KK Women’s and Children’s Hospital, Singapore, Singapore; ^6^Division of Critical Care Medicine, Department of Paediatrics, Duke University School of Medicine, Durham, NC, United States; ^7^Children’s Intensive Care Unit, KK Women’s and Children’s Hospital, Singapore, Singapore; ^8^Department of Emergency Medicine, KK Women’s and Children’s Hospital, Singapore, Singapore

**Keywords:** pediatrics, infant, mortality, infections, sepsis, global health

## Abstract

**Objective:**

Current knowledge on the global burden of infant sepsis is limited to population-level data. We aimed to summarize global case fatality rates (CFRs) of young infants with sepsis, stratified by gross national income (GNI) status and patient-level risk factors.

**Methods:**

We performed a systematic review and meta-analysis on CFRs among young infants < 90 days with sepsis. We searched PubMed, Cochrane Central, Embase, and Web of Science for studies published between January 2010 and September 2019. We obtained pooled CFRs estimates using the random effects model. We performed a univariate analysis at patient-level and a meta-regression to study the associations of gestational age, birth weight, onset of sepsis, GNI, age group and culture-proven sepsis with CFRs.

**Results:**

The search yielded 6314 publications, of which 240 studies (*N* = 437,796 patients) from 77 countries were included. Of 240 studies, 99 were conducted in high-income countries, 44 in upper-middle-income countries, 82 in lower-middle-income countries, 6 in low-income countries and 9 in multiple income-level countries. Overall pooled CFR was 18% (95% CI, 17–19%). The CFR was highest for low-income countries [25% (95% CI, 7–43%)], followed by lower-middle [25% (95% CI, 7–43%)], upper-middle [21% (95% CI, 18–24%)] and lowest for high-income countries [12% (95% CI, 11–13%)]. Factors associated with high CFRs included prematurity, low birth weight, age less than 28 days, early onset sepsis, hospital acquired infections and sepsis in middle- and low-income countries. Study setting in middle-income countries was an independent predictor of high CFRs. We found a widening disparity in CFRs between countries of different GNI over time.

**Conclusion:**

Young infant sepsis remains a major global health challenge. The widening disparity in young infant sepsis CFRs between GNI groups underscore the need to channel greater resources especially to the lower income regions.

**Systematic Review Registration:**

[www.crd.york.ac.uk/prospero], identifier [CRD42020164321].

## Introduction

Infant sepsis is an important public health challenge with a significant burden of disease. Globally, an estimated 1.3–3.9 million young infants experience sepsis and 400,000–700,000 die from sepsis-related conditions annually (1). In the Sub-Saharan African region alone, young infant sepsis incurs an economic burden of US$10 – $469 billion annually (2).

Sepsis remains a significant cause for death and accounts for up to 15% of all young infant deaths (3). One of the targets in the United Nations Sustainable Developmental Goals 3 (SDG 3) is to reduce neonatal mortality to 12 per 1,000 livebirths and under-5 mortality to 25 per 1,000 livebirths by 2030 (4, 5). The World Health Organization (WHO) has reported that an estimated 84% of young infant deaths due to sepsis are preventable (1). Reducing young infant sepsis mortality can contribute to achieving the SDG3 targets by 2030. Pediatric sepsis survivors are at higher risk of poor neurodevelopmental sequelae including neurocognitive deficits and developmental delay (6–8). A better understanding of young infant sepsis can provide valuable insights to inform strategies that span prevention, diagnosis and intervention to mitigate long term mortality and morbidity.

The recent population-based systematic review and meta-analysis on young infant sepsis mortality performed by Fleischmann et al. reviewed data from 1979 to 2019 and reported a population rate of 2824 (95% CI, 1892–4194) neonatal sepsis cases per 100,000 livebirths worldwide with a mortality of 17.6% (95% CI, 10.3–28.6%) (9). Sepsis incidence was reported to be highest in preterm and very low birth weight (VLBW) infants. However, this seminal study only included 14 countries with available population-level data with majority originating from middle-income countries. In addition, there is still a gap in mortality data in low-income countries. This limits the accurate estimate of the global burden of young infant sepsis (10). A comprehensive systematic review and meta-analysis on young infant sepsis case fatality rates (CFRs) from different income level countries will allow for a timely update to previously published literature on the global burden of young infant sepsis, and provide a more complete understanding on the global burden of young infant sepsis.

We therefore aimed to summarize global CFRs for young infants (<90 days) with sepsis, published from January 2010 to September 2019. We also aimed to describe any differences in young infant sepsis mortality among countries of different gross national income (GNI) status over time.

## Methods

We performed a systematic review and meta-analysis using the Preferred Reporting Items for Systematic Reviews and Meta-Analyses (PRISMA) 2020 guidelines (11). This study is registered with PROSPERO (CRD42020164321). The protocol is available online (12).

### Eligibility Criteria

We included randomized controlled trials (RCTs), cohort studies and cross-sectional studies that were published between January 2010 and September 2019. This range was chosen to provide an update on a previously published systematic review on the global burden of neonatal sepsis (10). We excluded studies published before January 2010 in view of the substantial changes in neonatal sepsis diagnosis and management over time.

We identified studies that contained the following specified elements of population, exposure, outcome and study design. We defined the population as infants less than 90 days old, regardless of gestational age. The neonatal period was defined as the first 28 postnatal days in term and post-term newborns, and day of birth through the expected date of delivery plus 27 days for preterm newborns (13). We chose to study infants less than 90 days old to obtain a comprehensive picture of sepsis burden, as serious infections in the young infant population can present past 28 postnatal days (14). If a study included both pediatric and adult population, it was included only if data pertaining to the infant population (<90 days) could be extracted. We defined the exposure as sepsis, which could be of bacterial, viral or fungal origin (15). We included viral infections because young infants can suffer from long term deficits from invasive viral illnesses (16, 17). Due to the lack of a gold standard for diagnosis of young infant sepsis (18), we decided to include all studies with sepsis as defined by study authors. However, we documented if the study defined sepsis according to the International Paediatric Sepsis Consensus Conference (15). We defined our primary outcome as the CFR, which was computed based on the number of deaths divided by the number of infants with sepsis.

We excluded case-control studies, case reports, animal studies, laboratory studies and publications that were not in English. We excluded studies with a primary focus on necrotising enterocolitis, respiratory distress syndrome without a primary sepsis study population, leukemia or other malignancies. We also excluded studies with a sample size of less than 50 to avoid small study effects (19).

### Information Sources and Search Strategy

We searched PubMed, Cochrane Central, Embase and Web of Science to identify eligible studies. The search was conducted on 17 September 2019 with a search strategy developed in consultation with research librarians experienced in systematic reviews and meta analyses. Strategic keywords used include “neonates,” “infants,” “sepsis,” “neonatal sepsis,” and “mortality.” The detailed search strategy can be found in Supplementary Table 1. We ensured that there were no completed or ongoing trials evaluating global burden of neonatal sepsis by searching PROSPERO, ClinicalTrials.gov, International Standard Randomized Controlled Trial Number (ISRCTN) registry, World Health Organization International Clinical Trials Registry Platform (ICTRP), and European Union Clinical Trials Register.

### Study Selection Process

Covidence (Australia) was used for the review of articles. Three reviewers (MG, BY, SS) independently conducted the database search and screened the title and abstracts for relevance, and subsequently assessed the full-text of shortlisted articles for eligibility. Any conflict on study eligibility were resolved in discussion with the senior author (S-LC). Reason(s) for exclusion of each article was (were) recorded.

### Data Collection Process and Data Items

Four reviewers (MG, BY, SS, and WL) independently carried out the data extraction using a standardized data collection form, and any conflict was resolved by discussion. Study variables included were study characteristics (e.g., study year, study design, geographical origin, sepsis definition, sample size), patient demographics (e.g., age, gender, gestational age, birth weight), patient characteristics (e.g., severity of sepsis, comorbidities, maternal risk factors, microbiological data, sources of infection, onset of sepsis, interventions, duration of hospital stay and blood markers), and outcome (deaths, timeframe to mortality). GNI was determined according to the World Bank Country Classification (20). We contacted the corresponding authors for any missing or unreported data via email. A second reminder email was sent 2 weeks later. When there was no reply 1 month from the first email we considered the team to be un-contactable.

### Study Risk of Bias Assessment

We assessed the risk of bias using the Cochrane risk-of-bias tool for RCTs (21), and the Newcastle-Ottawa Scale for all observational studies (22). Two assessors (SS and WL) independently carried out the assessment, and any conflict was resolved by discussion or with input from a third independent reviewer (MG). An overall rating of high, moderate or low risk was given to each study.

### Effect Measures and Synthesis Methods

Categorical variables were summarized as frequencies and percentages while continuous variables were summarized as means with standard deviations (SD). We generated pooled CFRs and the 95% confidence intervals (95% CI) using the DerSimonian and Laird method (23). We performed a univariate analysis at patient-level to assess the association between the CFRs and each variable (birth weight, age, gestational age, type of sepsis, source of infection either hospital or community acquired and culture-proven sepsis). We also performed a multivariable meta-regression at study level to assess the association between CFRs among infants less than 90 days and the following variables determined *a priori* – gestational age, birth weight, onset of sepsis, GNI, age group, and culture-proven sepsis. For each variable in the multivariable meta-regression analysis, only studies that exclusively looked at high risk groups [preterm infants, infants with low birth weight (LBW), early onset sepsis, countries with middle- and low-income, age <28 days and culture-proven sepsis] were selected. These studies were compared to reference groups of lower risk, and included studies that did not exclusively contain the high risk groups. For example, when studying the effect of birth weight, we compared studies that only included LBW and VLBW infants, as compared to studies that included infants of normal birth weight. For GNI status, we took high-income groups as the reference group. For study design, we took RCTs as the reference group.

All statistical analysis was done using Stata (v16.1, College Station, TX, United States). We used *I*^2^ statistics to quantify heterogeneity between studies. We performed two sensitivity analyses in which we included: (1) only studies with culture-proven sepsis; and (2) only studies with low risk of bias, evaluating CFRs and temporal trends limiting to these studies.

## Results

### Study Selection

**FIGURE 1:**
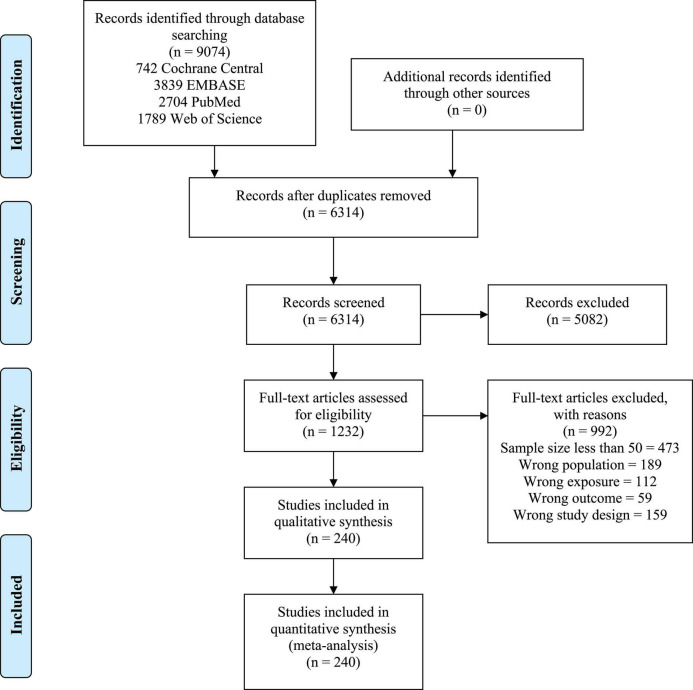
PRISMA flow diagram.

Among 6314 articles screened, 240 studies (with a total of 437,796 patients) met the inclusion criteria and were included for analysis (Figure 1).

### Study Characteristics

**FIGURE 2:**
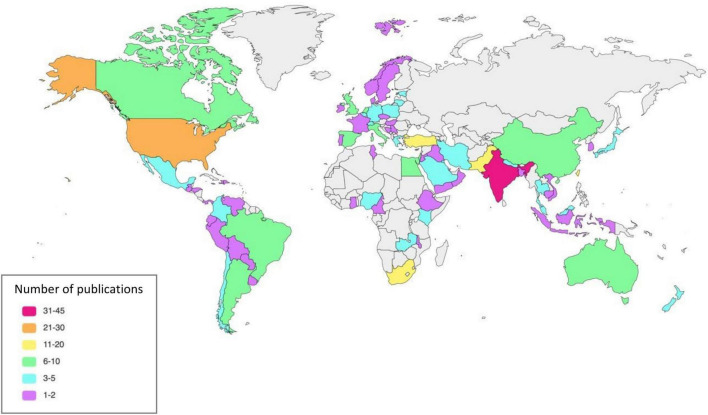
Publication by countries.

Characteristics of the included studies and study population are summarized in Tables 1, 2 and Supplementary Table 2. The studies originate from 77 countries and six continents (Figure 2).

**TABLE 1:** Characteristics of studies.

	High-income countries (*n* = 99)	Upper-middle-income countries (*n* = 44)	Lower-middle-income countries (*n* = 82)	Low-income countries (*n* = 6)	Multiple income level countries (*n* = 9)	All studies (*n* = 240)
**Study design**						
Cross sectional study, *n* (%)	3 (1)	5 (2)	11 (5)	1 (0.4)	2 (0.8)	22 (9)
Randomized controlled trial, *n* (%)	2 (0.8)	2 (0.8)	9 (4)	1 (0.4)	1 (0.4)	15 (6)
Prospective cohort study, *n* (%)	31 (13)	14 (6)	34 (14)	1 (0.4)	5 (2)	85 (35)
Retrospective cohort study, *n* (%)	63 (26)	23 (10)	27 (11)	3 (1)	1 (0.4)	117 (49)
**Continents** *						
Africa, *n* (%)	3 (1)	8 (3)	20 (8)	3 (1)	4 (2)	38 (16)
Asia, *n* (%)	33 (14)	23 (10)	70 (29)	1 (0.4)	6 (3)	133 (55)
Australia, *n* (%)	4 (2)	0	0	0	3 (1)	7 (3)
Europe, *n* (%)	34 (14)	13 (5)	2 (0.8)	0	4 (2)	53 (22)
North America, *n* (%)	28 (12)	4 (2)	0	2 (0.8)	6 (3)	40 (17)
South America, *n* (%)	0	7 (3)	0	0	6 (3)	13 (5)
**Studies that include culture-proven sepsis,** *n* (%)	91 (38)	40 (17)	74 (31)	4 (2)	6 (3)	215 (90)

*Some countries are transcontinental and hence total n for countries does not add up to 240.

**TABLE 2:** Characteristics of study population.

	High-income countries (*n* = 99)	Upper-middle-income countries (*n* = 44)	Lower-middle-income countries (*n* = 82)	Low-income countries (*n* = 6)	Multiple income level countries (*n* = 9)	All studies (*n* = 240)
**Age of patients**						
Age of patients, mean ± SD, days	27.9 ± 20.4	16.2 ± 15.6	9.8 ± 10.7	1.7	–	13.2 ± 15.5
Studies that exclusively studied neonates (<28 days), *n* (%)	55 (23)	31 (13)	70 (29)	5 (2)	3 (1)	164 (68)
**Birth weight of patients**						
Birth weight of patients, mean (SD), g	978 ± 356	1397 ± 522	2328 ± 666	–	–	1481 ± 517
Studies that exclusively studied low birth weight infants (<2500 g), *n* (%)	17 (7)	4 (2)	3 (1)	0	2 (0.8)	26 (11)
**Gestational age of patients**						
Gestational age, mean (SD), weeks	28.0 ± 3.0	29.8 ± 3.1	35.7 ± 2.9	–	37.0 ± 4.0	30.4 ± 3.0
Studies that exclusively studied preterm infants (<37 weeks), *n* (%)	13 (5)	6 (3)	5 (2)	0 (0)	0 (0)	24 (10)
**Types of sepsis**						
Studies that exclusively studied early onset sepsis (based on author’s definition), *n* (%)	15 (6)	3 (1)	7 (3)	0	1 (0.4)	26 (11)
**Predominant Causative Organisms**						
Gram-positive bacteria, *n* (%)	62 (26)	17 (7)	15 (6)	2 (0.8)	6 (3)	102 (43)
Gram-negative bacteria, *n* (%)	20 (8)	18 (8)	48 (20)	4 (2)	1 (0.4)	91 (38)
Viral, *n* (%)	1 (0.4)	0	0	0	0	1 (0.4)
Fungal, *n* (%)	6 (3)	2 (0.8)	3 (1)	0	1 (0.4)	12 (5)

The greatest number of studies were conducted in Asia with 133 studies (63042 patients), followed by Europe with 53 studies (23639 patients), North America (40 studies, 253786 patients), Africa (38 studies, 39554 patients), South America (13 studies, 160386 patients) and Australia (7 studies, 3270 patients).

Of the 240 studies, 99 (24–122) (41%) were conducted in high-income countries, 44 (123–166) (18%) in upper-middle-income countries, 82 (167–248) (34%) in lower-middle-income countries, and 6 (249–254) (3%) in low-income countries. Nine (255–263) (4%) studies were conducted in countries with multiple income levels.

Among all studies, 215/240 studies (90%) included culture-proven sepsis in their study population while 175/240 (73%) used a combination of clinical and laboratory criteria. 164/240 (68%) studies exclusively studied neonates (<28 days), 24/116 (21%) studies exclusively studied preterm infants, 26/125 (21%) exclusively studied LBW infants and 26/160 (16%) exclusively studied early onset sepsis. Out of 240 studies, 55 (23%) studies reported CFRs stratified by preterm versus term, 28 (12%) studies reported CFRs in community versus hospital acquired sepsis, 107 (45%) studies reported CFRs defined by early or late onset sepsis, and 56 (23%) studies reported birth weight related specific CFRs.

### Patient Characteristics

Overall mean age of the study population was 13.2 ± 15.5 days. Mean birth weight and gestational age of the study population was 1480 ± 516 grams and 30.4 ± 3.0 weeks, respectively.

One hundred and two studies (43%) reported gram positive organisms as the predominant organisms causing sepsis. Among the studies conducted in high-income countries, 62 of 99 studies (63%) reported gram positive organisms as the predominant causative organisms, while gram negative organisms were the predominant causative organisms in studies conducted in middle- or low-income countries – 18/44 (41%) in upper-middle-income countries, 48/82 (59%) in lower-middle-income countries, 4/6 (67%) in low-income countries.

**FIGURE 3:**
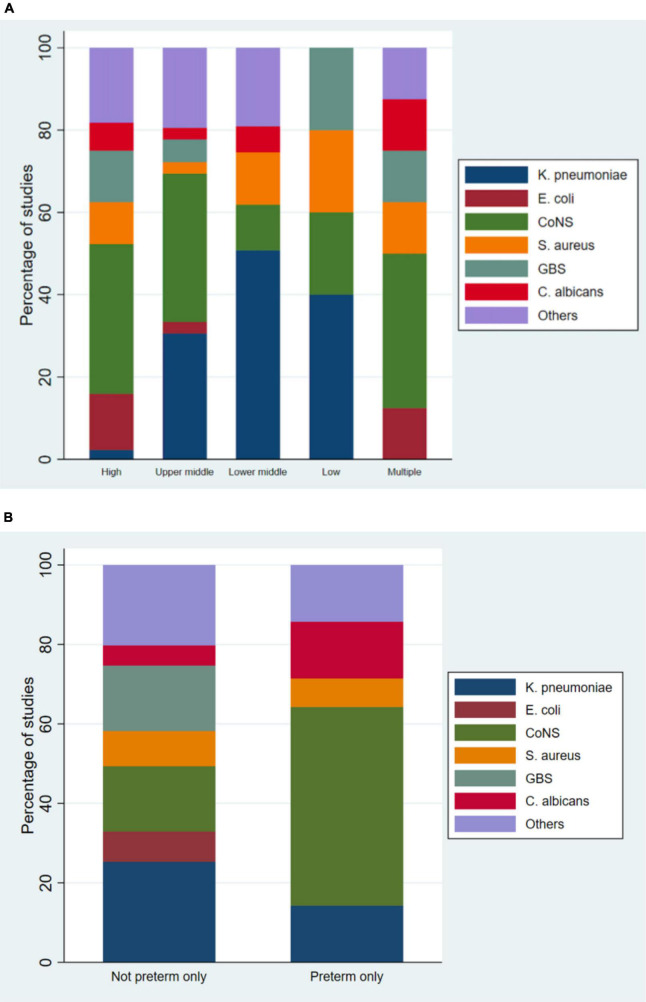
Causative organisms categorized by gross national income **(A)** and gestational age **(B)**.

Amongst the 200 studies that reported pathogens causing infant sepsis, the most common organisms reported were *Coagulase-negative* staphylococci (28%), followed by *Klebsiella pneumoniae* (24), *Staphylococcus aureus* (10%), *Group B Streptococcus* (8%), *Escherichia coli* (7%), and *Candida albicans* (6%). Among 24 studies that exclusively studied preterm infants, 21 studies reported the most common organism, in which the most common organism was coagulase-negative staphylococci, reported by 10/21 (48%) studies (Figure 3). Among 26 studies that exclusively studied LBW infants, 24 studies reported the most common organism, in which the most common organism was coagulase-negative staphylococci, reported by 13/24 (54%) studies.

### Outcomes

Overall, the pooled CFR was 18% (95% CI, 17–19%). The CFR was lowest for high-income countries [12% (95% CI, 11–13%)], as compared to upper-middle-income countries [21% (95% CI, 18–24%)] and lower-middle-income countries [24% (95% CI, 21–26%)]. Low-income countries had the highest CFR [25% (95% CI, 7–43%)]. Pooled CFR for continents was highest for Africa 24% (95% CI, 21–27%) and lowest in Australia 14% (95% CI, 10–18%) (Table 3). For study designs, cross sectional studies had a higher CFR of 22% (95% CI, 16–28%), while RCTs had a CFR of 13% (95% CI, 10–17%). Studies with a low risk of bias had a pooled CFR of 17% (95% CI, 16–18%).

**TABLE 3:** Pooled Case Fatality Rates based on study characteristics.

	Pooled CFRs (95% Confidence Interval)
**Gross National Income**	
Low-income countries (6)^†^	25% (7–43%)
Lower-middle-income countries (82)	24% (21–26%)
Upper-middle-income countries (44)	21% (18–24%)
High-income countries (99)	12% (11–13%)
**Study designs**	
Cross-sectional studies (22)	22% (16–28%)
Prospective studies (85)	20% (18–22%)
Retrospective studies (117)	17% (16–18%)
Randomized controlled trials (15)	13% (10–17%)
**Study continent**	
Africa (38)	24% (21–27%)
South America (13)	20% (17–22%)
Asia (133)	19% (18–21%)
North America (40)	18% (15–20%)
Europe (53)	16% (14–17%)
Australia (7)	14% (10–18%)
**Studies with low risk of bias** (184)	17% (16–18%)
**Overall pooled CFR**	18% (17–19%)

*^†^(N) refers to the number of studies that studied the variable and reported mortality.*

**FIGURE 4:**
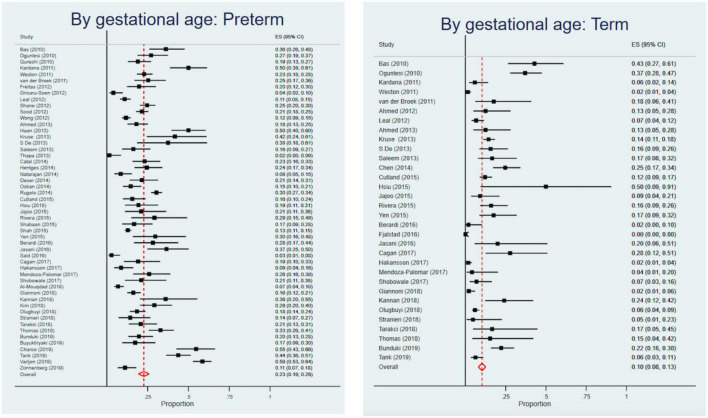
Forest plots of preterm and term Case Fatality Rates.

Among studies that reported CFRs by birth weight, the highest CFR was for VLBW infants [24% (95% CI, 21–26%] in comparison to LBW infants [23% (95% CI, 21–26%) and normal birth weight infants [15% (95% CI, 10–21%)]. Studies exclusively on neonates had a higher CFR of 18% (95% CI, 17–19%) compared to studies that included older infants [15% (95% CI, 14–17%)]. Studies on preterm infants had a CFR of 23% (95% CI, 19–26%), more than double compared to term infants with a CFR of 10% (95% CI, 8–13%) (Figure 4). Infants with early onset sepsis (<72 h of life) had a CFR of 20% (95% CI, 17–24%), while infants with early and late onset sepsis had a CFR of 16% (95% CI, 14–18%). Infants with hospital-acquired infections had a CFR of 23% (95% CI, 17–29%), higher than those with community-acquired infections [14% (95% CI, 9–19%)]. Infants with culture- proven sepsis had a CFR of 20% (95% CI, 19–22%) (Table 4).

**TABLE 4:** Pooled Case Fatality Rates for study population characteristics.

	Pooled CFRs (95% Confidence Interval)
**Birth weight**	
<1500 g (45)^‡^	24% (21–26%)
<2500 g (51)	23% (21–26%)
≥2500 g (16)	15% (10–21%)
**Age**	
Studies that exclusively studied neonates < 28 days (164)	18% (17–19%)
**Gestational age**	
Preterm (52)	23% (19–26%)
Term (32)	10% (8–13%)
**Type of sepsis**	
Early onset (<72 h) (44)	20% (17–24%)
All (both) (83)	16% (14–18%)
**Source of infection**	
Hospital acquired (22)	23% (17–29%)
Community acquired (14)	14% (9–19%)
**Culture-proven sepsis** (178)	20% (19–22%)
**Overall pooled case fatality rate** (240)	18% (17–19%)

*^‡^(N) refers to the number of studies that studied the variable and reported mortality.*

**FIGURE 5:**
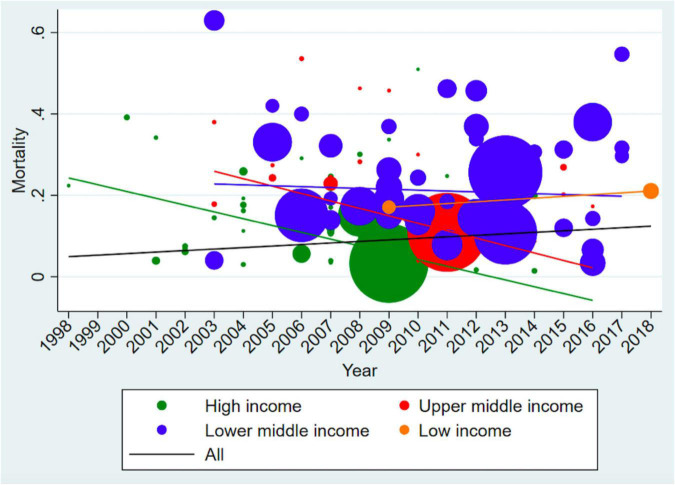
Time trend analysis of Case Fatality Rates (including only low risk of bias studies). The size of the bubble is proportionate to the number of infants in the study, while the line represents the trends of case fatality rates over time. There is an increasing trend for low-income countries and decreasing trend for middle- and high-income countries overtime. The overall trend for young infant sepsis case fatality rates is increasing.

There was an overall increasing trend in young infant sepsis CFRs over time (Figure 5 and Supplementary Figure 1). When annual pooled CFRs were stratified according to GNI status, there was a widening of the disparity between GNI and young infant CFRs, with time. This is consistent with the sensitivity analysis including only studies with low risk of bias (Figure 5).

### Meta Regression

Nine (4%) studies that were conducted in countries with multiple income levels were excluded from the analysis. Independent predictors of higher CFRs include upper-middle- and lower-middle-income countries [regression coefficient 0.12 (95% CI, 0.04–0.21); *p* = 0.003 and 0.14 (95% CI, 0.06–0.22); *p* = 0.001 respectively] (Table 5).

**TABLE 5:** Meta regression predicting Case Fatality Rates among infants less than 90 days.

Characteristics	Univariate analysis		Multivariable analysis	
	Coefficient (95% CI)	*P*-value	Coefficient (95% CI)	*P*-value
**Gestational age**§				
Term	1 [Reference]	NA	1 [Reference]	NA
Preterm	0.04 (–0.01, 0.09)	0.11	0.11 (–0.02, 0.23)	0.09
**Birth weight** **				
Normal birth weight	1 [Reference]	NA	1 [Reference]	NA
Low birth weight	0.08 (0.03, 0.12)	0.001	–0.02 (–0.16, 0.13)	0.83
**Type of sepsis** ^††^				
Late onset sepsis	1 [Reference]	NA	1 [Reference]	NA
Early onset sepsis	0.0005 (–0.07, 0.07)	0.99	0.05 (–0.05, 0.15)	0.35
Both early and late onset sepsis	0.05 (–0.006, 0.10)	0.08	0.04 (–0.04, 0.12)	0.33
**Gross national income**				
High-income	1 [Reference]	NA	1 [Reference]	NA
Upper-middle-income	0.05 (0.03, 0.07)	<0.001	0.12 (0.04, 0.21)	0.003
Lower-middle-income	0.15 (0.11, 0.19)	<0.001	0.14 (0.06, 0.22)	0.001
Low-income	0.20 (0.08, 0.31)	0.001	0.04 (–0.13, 0.21)	0.65
**Age < 28 days**	–0.06 (–0.08, –0.05)	<0.001	–0.06 (–0.15, 0.03)	0.17
**Culture-proven sepsis**	0.07 (0.04, 0.09)	<0.001	0.08 (–0.04, 0.20)	0.20

*§Term is defined as 37 weeks or more, preterm is defined as less than 37 weeks.*

**Normal birthweight is defined as ≥ 2500 g, low birthweight is defined as < 2500 g.

*^††^Early onset sepsis is defined as sepsis onset < 72 h.*

Heterogeneity among studies were high with *I*^2^ ranging 91–99%. This was persistent even with our sensitivity analyses that included only studies with culture-proven sepsis, and only studies with low risk of bias.

### Reporting Biases

Five (33%) of the 15 RCTs and 179 (80%) of the 225 observational studies were assigned low risk of bias respectively (Supplementary Tables 3, 4). RCTs that were assigned moderate or high risk of bias were mainly due to the lack of blinding of participants or outcome assessors. Observational studies that were assigned moderate or high risk of bias were mainly due to either undefined or poorly defined clinical sepsis criteria, lack of a sufficient duration of follow up for assessing mortality or lack of adequate follow up rate.

## Discussion

In this systematic review and meta-analysis, we provide an update to previously published literature on the global burden of young infant sepsis. To the best of our knowledge, this is the largest systematic review and meta-analysis of young infant sepsis CFRs, comprising 240 studies from 77 countries over six continents, which are not limited to population-based studies. Of the 240 studies included, 132 (55%) studies from 36 countries were conducted in middle and low-income countries, compared to 22 studies from 11 countries that were included in the prior systematic review by Fleischmann et al. (9). Our data bridge the knowledge gap on young infant sepsis particularly from low- and middle-income countries. This is also the first study that reflects global young infant sepsis CFRs over time stratified by GNI – with a decreasing trend in high-income countries but a worrying increasing trend in low-income countries, providing new insights into global young infant sepsis CFR trajectories.

We found a widening disparity in young infant sepsis outcomes between countries of different GNI. Our study revealed that Africa with a majority of low-income countries bore the highest burden of young infant sepsis with a CFR of 24% compared to Europe and Australia with a CFR burden of 16 and 14% respectively. The WHO Global Report on the Epidemiology and Burden of Sepsis 2020 showed similar results where low- and middle-income countries shoulder a higher incidence and mortality burden of young infant sepsis, particularly in Sub-Saharan Africa and South East Asia (1). Fleischmann-Struzek et al. also reported that young infant sepsis mortality rates were two times higher in middle-income countries than high-income countries (10). Young infants from lower middle- and low-income countries often present with comorbidities such as malnutrition and dehydration that can increase the risk of severe sepsis and death (264). Furthermore, unrecognized or untreated perinatal infections increase the risk of young infant sepsis (264). Limited access to high-quality healthcare results in delays to diagnosis and treatment of young infant sepsis (265–267). This includes the lack of adequately equipped and staffed healthcare institutions (19), and insufficient subsidized healthcare services for the poor (268).

There is currently limited epidemiological data on infant sepsis, especially in low-middle- and low-income countries (9, 10). The WHO has repeatedly called for a global effort to generate sepsis epidemiological data, especially in low-income countries (1). This requires early and accurate diagnosis of sepsis, as well as an improved coordination between like-minded groups doing research in these countries. Systems-based changes to strengthen each country’s health system should target the achievement of universal health coverage (269). This will ensure the availability of essential health-care services that are safe, effective and affordable to all in low-income countries (270). With a global commitment to robust infant sepsis surveillance, universal health coverage and greater resources channeled to low-income countries, we can work toward reducing young infant CFRs globally, especially in low-income countries.

Our overall CFR of 18% for young infants with sepsis was consistent with prior reports (11–29%) (9, 10). Of potential concern is that our time trend analysis of young infant sepsis CFRs showed an overall increasing trend over time from 1998 to 2018. In contrast, the Global Burden of Disease study 2019 showed a decreasing trend of –11.5% (95% CI, –23.6% to –4.3%) change in deaths due to neonatal sepsis from 2009 to 2019 (271). The limited number of studies from middle- and low-income countries represented in the earlier time period in our meta-analysis could have resulted in falsely low global young infant CFRs. Notably, all studies with a reference year prior to 2002 (Figure 5) were conducted in high-income countries. In addition, a global shift in focus to better understand young infant sepsis epidemiology in recent years could have resulted in an increased reporting of young infant sepsis mortality in lower-middle- and low-income countries (1, 272), contributing to the increasing trend of young infant CFRs in the lower-middle- and low-income countries, and also the overall trend.

Our study highlighted several factors associated with higher CFRs in young infant sepsis, namely prematurity, LBW, age less than 28 days, early onset sepsis and hospital acquired infections and sepsis in middle- and low-income countries. Previous literature similarly demonstrated prematurity and LBW to be major risk factors of young infant sepsis (273). In addition, neonates aged less than 28 days reported higher CFRs. This could be attributed to a relatively immature immune system resulting in neonates being more susceptible to infections (274). Studies have also shown that early onset sepsis disproportionately affects preterm infants who are already at a higher risk for mortality, and can result in fulminant, multisystemic infections (275). Infants who develop hospital acquired infections often have lower birth weights, lower gestational age and greater comorbidities compared to community acquired infections (37, 276). This translates to lower functional reserves and decreased immunity defenses, resulting in infants who develop hospital acquired infections having more severe infections and poorer outcomes.

Our study focused on the association between geographical gross income status and CFRs. Nonetheless, we recognize the impact of social determinants of health on young infant sepsis CFRs. A previous study reported that patients of lower socioeconomic status and those who paid out-of-pocket (as compared to privately insured patients) experienced a greater risk of young infant sepsis mortality (39). In another study, it was reported that young infants born to mothers with lower education experienced higher young infant sepsis death rate (101). Young infants of non-White ethnicity have been reported to have higher odds ratio of young infant sepsis mortality as compared to infants of White ethnicity (109). Currently, few studies detail the impact of specific social determinants of health on young infant sepsis incidence and CFRs. Further research in this area may be helpful in informing strategies regarding resource distribution and utilization.

Our attempt to provide a global scope on young infant sepsis with an inclusion criteria that accepted a wide range of study populations and sepsis definition may have led to considerable heterogeneity (1). This is likely to be contributed by the differing underlying parameters used in each study – such as differences in study design, study period, geographical region as well as the predominant organisms. Previous studies have shown that meta-analyses addressing broad review questions may result in highly heterogenous studies (277). We generated pooled CFRs using the random effects model which accounts for total study variation including between-study variations. Nevertheless, we recognize wide variation between study populations, social determinants of health, resource availability and outcome measures used, so much so that the high heterogeneity limited precise answers on sepsis mortality among young infants (277).

### Limitations

Firstly, due to the lack of a gold standard for diagnosis of young infant sepsis, we accepted a wide range of sepsis definitions but categorized them into clinical, laboratory or culture-proven (18). Future studies utilizing a standardized sepsis definition that can be widely applied to all regions will facilitate comparative studies. Secondly, there was considerable heterogeneity in our meta-analysis, ranging from 91 to 99%. A sensitivity analysis including only studies with low risk of bias revealed similar results. We attributed this to variability in study populations, availability and delivery of health services, and outcome measures used. Thirdly, we excluded non-English studies which may have resulted in us missing useful data. We also excluded any studies with sample size less than 50 to reduce small study effects (19), which may have resulted in relevant studies being excluded. Finally, a lack of studies from the low- and middle-income countries in the earlier time period resulted in limited interpretation of the overall trend of young infant sepsis.

## Conclusion

Our review showed an overall global burden of young infant sepsis CFR of 18%, with an increasing disparity between low- and middle-income countries compared to high-income countries over time. Factors associated with higher CFRs included prematurity, LBW, age less than 28 days, early onset sepsis, hospital acquired infections and sepsis in middle- and low-income countries, of which sepsis in middle-income countries was an independent predictor of higher CFRs. The findings from our study serve as a global call to action to achieve further reductions in young infant sepsis mortality. Future initiatives should focus on improving the delivery of care to infants at higher risk of sepsis, especially in the low- and middle-income countries.

## Data Availability Statement

The original contributions presented in the study are included in the article/Supplementary Material, further inquiries can be directed to the corresponding author.

## Author Contributions

MG, WL, BY, and S-LC coordinated the study. MG, BY, JP, BT, and S-LC developed the search strategy and registered the protocol. MG, WL, BY, SS, and BT reviewed the studies and extracted data from the studies. WL and SS conducted risk of bias assessments. RG conducted the statistical analyses. MG, WL, RG, CH, JL, and S-LC performed the data interpretation. MG and WL wrote the original draft of the manuscript with revision from BY and SS. BY, SS, RG, JP, BT, CH, JL, and S-LC helped to revise the manuscript. All authors read and approved the final manuscript.

## Conflict of Interest

The authors declare that the research was conducted in the absence of any commercial or financial relationships that could be construed as a potential conflict of interest.

## Publisher’s Note

All claims expressed in this article are solely those of the authors and do not necessarily represent those of their affiliated organizations, or those of the publisher, the editors and the reviewers. Any product that may be evaluated in this article, or claim that may be made by its manufacturer, is not guaranteed or endorsed by the publisher.
