# Supplementary material for: Contemporary Trends in Global Mortality of Sepsis Among Young Infants Less Than 90 Days: A Systematic Review and Meta-Analysis
Source: Front Pediatr. 2022 Jun 3;10:890767. doi: 10.3389/fped.2022.890767 (PMC9204066; doi:10.3389/fped.2022.890767)
Supplement: Supplementary file 1 [file Presentation_1.pdf]

Supplementary File for Global Young Infant Sepsis Mortality

Supplementary Figure 1. Time trend analysis of Case Fatality Rates (including all studies)*

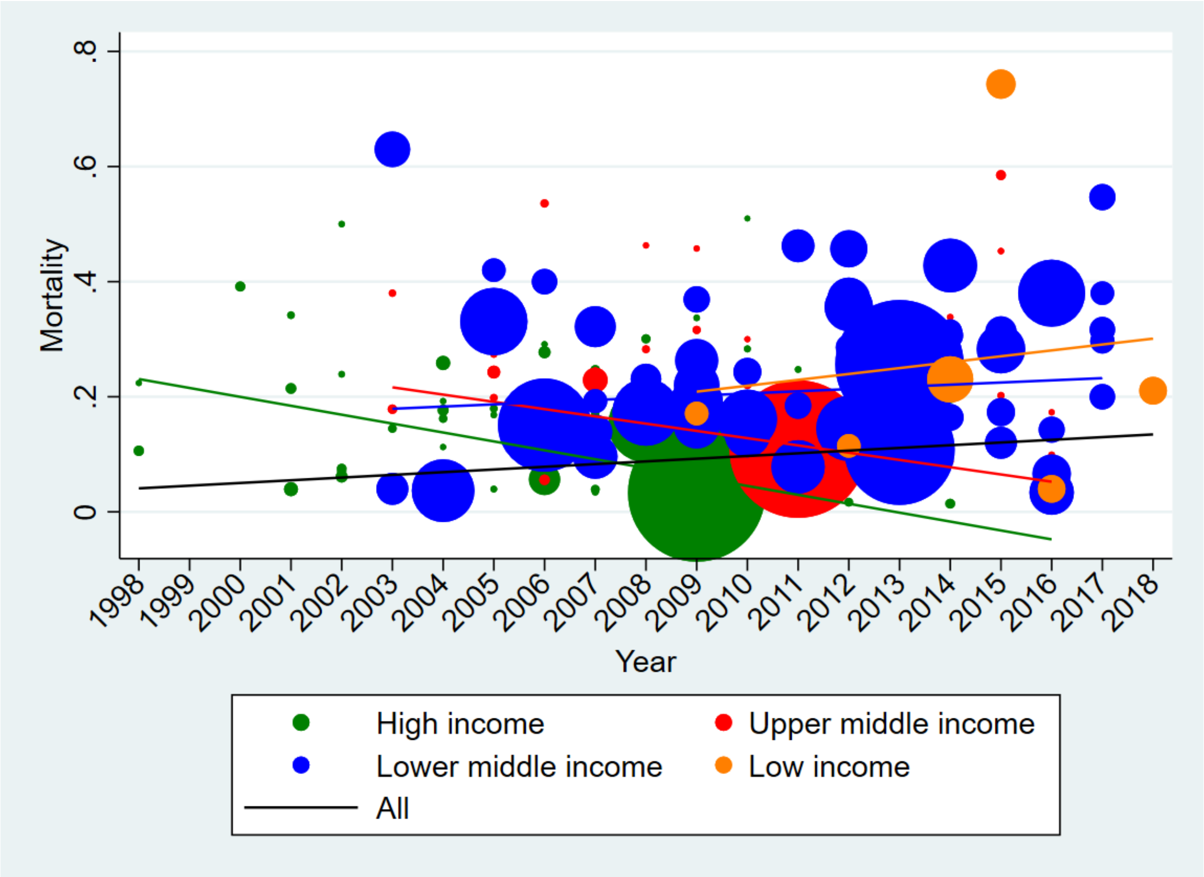

* There is an increasing trend for low- and lower-middle-income countries and decreasing trend for upper-middle- and high-income countries overtime. The overall trend for young infant sepsis case fatality rates is increasing.

Supplementary Figure 2. Forest plots of birth weight, onset of sepsis and source of sepsis Case Fatality Rates

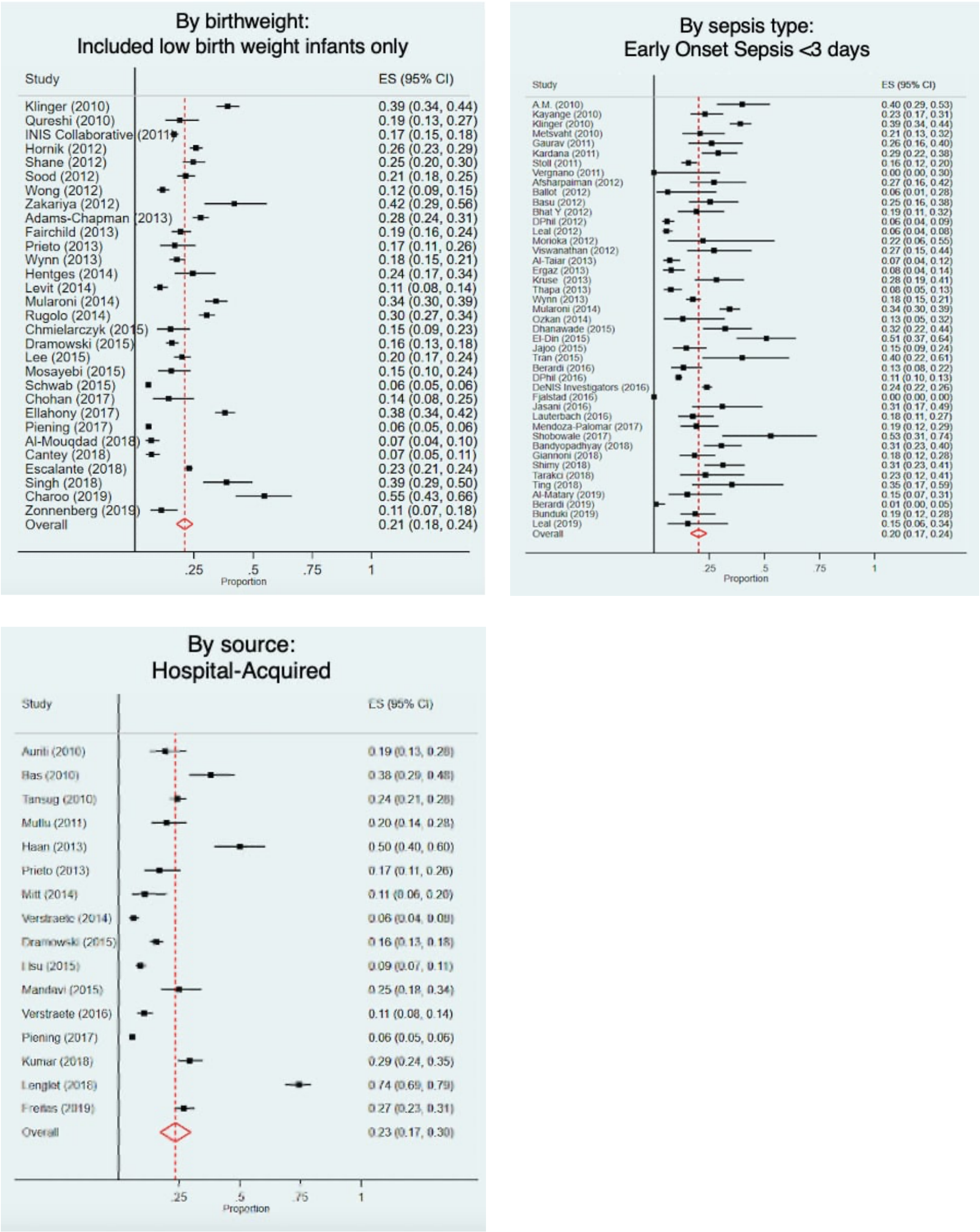

Supplementary Table 1. Search strategy

Cochrane Central	#1 MeSH descriptor: [Sepsis] this term only<br>#2 MeSH descriptor: [Shock, Septic] explode all trees<br>#3 (Pyemia OR Pyemias OR Pyohemia OR Pyohemias OR Pyaemia OR Pyaemias OR Septicemia OR Septicemias OR Blood Poisoning OR Blood Poisonings OR Sepsis OR Sepses OR Septic OR Endotoxemia):ti<br>#4 (#1 or #2 or #3)<br>#5 MeSH descriptor: [Infant] explode all trees<br>#6 (newborn OR newborns OR new born OR new borns OR newly born OR baby OR babies OR premature OR prematurity OR preterm OR pre term OR low birth weight OR low birthweight OR VLBW OR LBW OR infant OR infants OR infantile OR neonate OR neonates OR neonatal):ti<br>#7 (#5 or #6)<br>#8 (#4 and #7)<br>#9 MeSH descriptor: [Neonatal Sepsis] explode all trees<br>#10 (Neonatal Sepsis OR Neonatal Sepses OR Neonatal Late-Onset Sepsis OR Neonatal Late-Onset Sepses OR Neonatal Late Onset Sepsis OR Neonatal Late Onset Sepses OR Neonatal Early-Onset Sepsis OR Neonatal Early-Onset Sepses OR Neonatal Early Onset Sepsis OR Neonatal Early Onset Sepses):ti,ab,kw<br>#11 (#9 or #10)<br>#12 (#8 or #11)<br>#13 MeSH descriptor: [Mortality] explode all trees<br>#14 MeSH descriptor: [Infant Death] explode all trees<br>#15 (Mortality OR Mortalities OR fatal OR fatality OR fatalities OR death OR deaths OR survival OR demise):ti,ab,kw<br>#16 (#13 or #14 or #15)<br>#17 (#12 and #16)
EMBASE	#1 'sepsis'/de OR 'septic shock'/exp<br>#2 'infant'/exp<br>#3 pyemia:ti OR pyemias:ti OR pyohemia:ti OR pyohemias:ti OR pyaemia:ti OR pyaemias:ti OR septicemia:ti OR septicemias:ti OR 'blood poisoning':ti OR 'blood poisonings':ti OR sepsis:ti OR sepses:ti OR septic:ti OR endotoxemia:ti<br>#4 newborn:ti OR newborns:ti OR 'new born':ti OR 'new borns':ti OR 'newly born':ti OR baby:ti OR babies:ti OR premature:ti OR prematurity:ti OR preterm:ti OR 'pre term':ti OR 'low birth weight':ti OR 'low birthweight':ti OR vlbw:ti OR lbw:ti OR infant:ti OR infants:ti OR infantile:ti OR neonate:ti OR neonates:ti OR neonatal:ti<br>#5 (#1 AND #2)<br>#6 #3 NEAR/3 #4<br>#7 'newborn sepsis'/exp OR 'neonatal sepsis':ab,ti OR 'neonatal sepses':ab,ti OR 'neonatal late-onset sepsis':ab,ti OR 'neonatal late-onset sepses':ab,ti OR 'neonatal late onset sepsis':ab,ti OR 'neonatal late onset sepses':ab,ti OR 'neonatal early-onset sepsis':ab,ti OR 'neonatal early-onset sepses':ab,ti OR 'neonatal early onset sepsis':ab,ti OR 'neonatal early onset sepses':ab,ti<br>#8 (#5 OR #6 OR #7)<br>#9 'mortality'/exp OR 'child death'/exp OR mortality:ab,ti OR mortalities:ab,ti OR fatal:ab,ti OR fatality:ab,ti OR fatalities:ab,ti OR death:ab,ti OR deaths:ab,ti OR survival:ab,ti OR demise:ab,ti<br>#10 (#8 AND #9)
PubMed	#1 ((("Sepsis"[Mesh:NoExp] OR "Shock, Septic"[Mesh])) OR ((Pyemia[Title] OR Pyemias[Title] OR Pyohemia[Title] OR Pyohemias[Title] OR Pyaemia[Title] OR Pyaemias[Title] OR Septicemia[Title] OR Septicemias[Title] OR Blood Poisoning[Title] OR Blood Poisonings[Title] OR Sepsis[Title] OR Sepses[Title] OR Septic[Title] OR Endotoxemia[Title]))<br>#2 ("Infant"[Mesh]) OR ((newborn[Title] OR newborns[Title] OR new born[Title] OR new borns[Title] OR newly born[Title] OR baby[Title] OR babies[Title] OR premature[Title] OR prematurity[Title] OR preterm[Title] OR pre term[Title] OR low birth weight[Title] OR low birthweight[Title] OR VLBW[Title] OR LBW[Title] OR infant[Title] OR infants[Title] OR infantile[Title] OR neonate[Title] OR neonates[Title] OR neonatal[Title]))<br>#3 ("neonatal sepsis"[MeSH]) OR ((Neonatal Sepsis[Title/Abstract] OR Neonatal Sepses[Title/Abstract] OR Neonatal Late-Onset Sepsis[Title/Abstract] OR Neonatal Late-

	Onset Sepses[Title/Abstract] OR Neonatal Late Onset Sepsis[Title/Abstract] OR Neonatal Late Onset Sepses[Title/Abstract] OR Neonatal Early-Onset Sepsis[Title/Abstract] OR Neonatal Early-Onset Sepses[Title/Abstract] OR Neonatal Early Onset Sepsis[Title/Abstract] OR Neonatal Early Onset Sepses[Title/Abstract]))<br>#4 (#1 and #2)<br>#5 (#3 or #4)<br>#6 ((("mortality"[MeSH] OR "Infant Death"[Mesh]))) OR ((Mortality[Title/Abstract] OR Mortalities[Title/Abstract] OR fatal[Title/Abstract] OR fatality[Title/Abstract] OR fatalities[Title/Abstract] OR death[Title/Abstract] OR deaths[Title/Abstract] OR survival[Title/Abstract] OR demise[Title/Abstract]))<br>#7 (#5 and #6)
Web of Science	#1 TS=(Pyemia OR Pyemias OR Pyohemia OR Pyohemias OR Pyaemia OR Pyaemias OR Septicemia OR Septicemias OR "Blood Poisoning" OR "Blood Poisonings" OR Sepsis OR Sepses OR Septic OR Endotoxemia)<br>#2 TS=(newborn OR newborns OR "new born" OR "new borns" OR "newly born" OR baby OR babies OR premature OR prematurity OR preterm OR "pre term" OR "low birth weight" OR "low birthweight" OR VLBW OR LBW OR infant OR infants OR infantile OR neonate OR neonates OR neonatal)<br>#3 TS=("Neonatal Sepsis" OR "Neonatal Sepses" OR "Neonatal Late-Onset Sepsis" OR "Neonatal Late-Onset Sepses" OR "Neonatal Late Onset Sepsis" OR "Neonatal Late Onset Sepses" OR "Neonatal Early-Onset Sepsis" OR "Neonatal Early-Onset Sepses" OR "Neonatal Early Onset Sepsis" OR "Neonatal Early Onset Sepses")<br>#4 TS=((Pyemia OR Pyemias OR Pyohemia OR Pyohemias OR Pyaemia OR Pyaemias OR Septicemia OR Septicemias OR "Blood Poisoning" OR "Blood Poisonings" OR Sepsis OR Sepses OR Septic OR Endotoxemia) NEAR/3 (newborn OR newborns OR "new born" OR "new borns" OR "newly born" OR baby OR babies OR premature OR prematurity OR preterm OR "pre term" OR "low birth weight" OR "low birthweight" OR VLBW OR LBW OR infant OR infants OR infantile OR neonate OR neonates OR neonatal))<br>#5 (#3 OR #4)<br>#6 TS=(Mortality OR Mortalities OR fatal OR fatality OR fatalities OR death OR deaths OR survival OR demise)<br>#7 (#5 AND #6)

Supplementary Table 2. Summary of included studies

Income group	Author and publication year	Country	Sepsis definition	N	Mortality
High	Al-Matary, 2019	Saudi Arabia	Clinical + Labs	245	29
High	Singh, 2019	Multi	Labs	454	66
High	Pruitt, 2019	USA	Clinical + Labs	350	5
High	Berardi, 2019	Italy	Labs	108	1
High	Glikman, 2019	Israel	Labs	549	66
High	Al Luhidan, 2019	Saudi Arabia	Clinical + Labs	55	2
High	Zonnenberg, 2019	Netherlands	Clinical + Labs	117	13
High	Abdellatif, 2019	Oman	Clinical + Labs	125	11
High	Hamdy, 2019	USA	Clinical + Labs	78	7
High	Hsu, 2018	Taiwan	Clinical + Labs	113	32
High	Ting, 2018	Canada	Labs	286	86
High	Cantey, 2018	USA	Labs	285	20
High	Giannoni, 2018	Switzerland	Goldstein et al	444	52
High	Bohanon, 2018	USA	Clinical + Labs	160667	5291
High	Al-Mouqdad, 2018	Saudi Arabia	Clinical + Labs	291	20
High	Kim, 2018	South Korea	Clinical + Labs	79	23
High	Benedict, 2018	USA	Clinical + Labs	90	14
High	Glikman, 2018	Israel	Labs	255	10
High	Kuzniewicz, 2017	USA	Labs	51	2
High	Chen, 2017	Taiwan	Clinical + Labs	144	8
High	Blanchard, 2017	Canada	Labs	89	25
High	Carr, 2017	Australia	Labs	332	25
High	Agyeman, 2017	Switzerland	Goldstein et al	548	55
High	Mendoza-Palomar, 2017	Spain	Clinical + Labs	78	15
High	Wu, 2017	Taiwan	Clinical + Labs	781	53
High	Gowda , 2017	Australia	Clinical + Labs	133	8
High	Hakansson, 2017	Sweden	Clinical + Labs	395	13
High	Ree, 2017	Netherlands	Clinical + Labs	460	24
High	Drageset, 2017	Multi	Clinical + Labs	238	4
High	Piening, 2017	Germany	Clinical + Labs	4094	234
High	Sanchez-Pinto, 2017	USA	Clinical + Labs	178	21
High	Bulkowstein, 2016	Israel	Clinical + Labs	184	20
High	Fjalstad, 2016	Norway	Clinical + Labs	1538	1
High	DPhil, 2016	USA	Labs	1484	165
High	Pugni, 2016	Italy	Goldstein et al	51	26

High	Ivady, 2016	Hungary	Clinical + Labs	59	5
High	Verstraete, 2016	Belgium	Labs	342	36
High	Tsai, 2016	Taiwan	Clinical + Labs	749	90
High	Said, 2016	France	Clinical + Labs	105	3
High	Lauterbach, 2016	Poland	Clinical + Labs	458	19
High	Deshpande, 2016	Canada	Clinical + Labs	119	19
High	Berardi, 2016	Italy	Labs	90	12
High	Lee, 2015	South Korea	Clinical + Labs	504	100
High	Chmielarczyk, 2015	Poland	Clinical + Labs	100	15
High	Tsai, 2015	Taiwan	Clinical + Labs	942	68
High	Lai, 2015	Taiwan	Clinical + Labs	772	73
High	Yen, 2015	Taiwan	Clinical + Labs	67	15
High	Hsu, 2015	Taiwan	Clinical + Labs	715	64
High	Schwab, 2015	Germany	Clinical + Labs	6911	390
High	Hsiu, 2015	Taiwan	Clinical + Labs	59	12
High	Bergin, 2015	USA	Labs	258	18
High	Mitsiakos, 2015	Greece	Labs	81	20
High	Shah, 2015	Canada	Clinical + Labs	1104	142
High	Cobos-Carrascosa, 2015	Spain	Labs	71	8
High	Schlapbach , 2015	Multi	Goldstein et al	510	59
High	Levit, 2014	USA	Clinical + Labs	424	45
High	Chen, 2014	China	Goldstein et al	97	24
High	Bizzarro, 2014	USA	Clinical + Labs	158	54
High	Lutsar, 2014	Multi	Goldstein et al	113	9
High	Verstraete, 2014	Belgium	Clinical + Labs	620	38
High	Tsai, 2014	Taiwan	Clinical + Labs	713	68
High	Chu, 2014	Taiwan	Clinical + Labs	769	82
High	Tsai, 2014	Taiwan	Clinical + Labs	333	52
High	Natarajan, 2014	USA	Clinical + Labs	106	9
High	Mitt, 2014	Estonia	Clinical + Labs	74	8
High	Oeser, 2014	United Kingdom	Labs	84	18
High	Balamuth, 2014	USA	Clinical + Labs	46339	7033
High	Dolapo, 2014	USA	Clinical	156	28
High	Bamberger, 2014	Israel	Labs	52	5
High	Ahmed, 2013	Qatar	Labs	176	30
High	Wynn, 2013	USA	Labs	504	89
High	Haan, 2013	Netherlands	Clinical + Labs	84	42
High	Adams-Chapman, 2013	USA	Labs	656	182

High	Ergaz, 2013	Israel	Clinical + Labs	991	39
High	Hammoud, 2013	Kuwait	Labs	89	30
High	Fairchild, 2013	USA	Labs	352	68
High	Luthander, 2013	Sweden	Clinical + Labs	221	32
High	Prieto, 2013	Spain	Clinical + Labs	95	16
High	Tsai, 2012	Taiwan	Clinical + Labs	234	7
High	Morioka, 2012	Japan	Clinical + Labs	51	10
High	Sood, 2012	USA	Labs	457	98
High	Hornik, 2012	USA	Labs	1032	267
High	Ahmed, 2012	Qatar	Labs	176	30
High	Hammoud, 2012	Kuwait	Clinical + Labs	949	111
High	Livorsi, 2012	USA	Labs	216	35
High	Grisaru-Soen, 2012	Israel	Labs	101	4
High	Villa, 2012	Spain	Labs	50	6
High	Shane, 2012	USA	Labs	316	78
High	Wong, 2012	Canada	Labs	582	68
High	Vergnano, 2011	United Kingdom	Labs	116	4
High	Stoll, 2011	USA	Labs	389	61
High	van der Broek, 2011	Netherlands	Labs	92	22
High	Schlapbach, 2011	Switzerland	Clinical + Labs	400	7
High	Al-Taiar, 2011	Kuwait	Clinical + Labs	153	19
High	Weston, 2011	USA	Labs	658	72
High	Metsvaht, 2010	Estonia	Clinical + Labs	67	14
High	Guilbert, 2010	France	Labs	220	39
High	Klinger, 2010	Israel	Clinical + Labs	383	150
High	Auriti, 2010	Italy	Clinical + Labs	104	20
Upper Middle	Varljen, 2019	Serbia	Clinical + Labs	282	165
Upper Middle	Buyuktiryaki, 2019	Turkey	Labs	52	9
Upper Middle	Macooie, 2019	Iran	Clinical + Labs	50	15
Upper Middle	Freitas, 2019	Brazil	Clinical + Labs	435	117
Upper Middle	Celik , 2019	Turkey	Clinical + Labs	141	55
Upper Middle	Leal, 2019	Mexico	Goldstein et al	50	5
Upper Middle	Stranieri, 2018	Brazil	Goldstein et al	70	8
Upper Middle	Olugbuyi, 2018	Jamaica	Clinical + Labs	691	70
Upper Middle	Yusef, 2018	Jordan	Clinical + Labs	68	23
Upper Middle	Escalante, 2018	Multi	Labs	3066	702
Upper Middle	Tarakci, 2018	Turkey	Clinical + Labs	94	19
Upper Middle	Thomas, 2018	South Africa	Clinical + Labs	155	49
Upper Middle	Neira, 2018	Brazil	Clinical + Labs	121842	13289

Upper Middle	Softic, 2017	Bosnia and Herzegovina	Clinical + Labs	136	25
Upper Middle	Siavashi, 2017	Iran	Clinical + Labs	81	8
Upper Middle	Fu, 2017	China	Labs	69	6
Upper Middle	Cagan, 2017	Turkey	Clinical + Labs	65	14
Upper Middle	Zhou, 2016	China	Clinical + Labs	64	29
Upper Middle	Arizaga-Ballesteros, 2015	Mexico	Goldstein et al	71	5
Upper Middle	Mosayebi, 2015	Iran	Clinical + Labs	104	16
Upper Middle	Turhan, 2015	Turkey	Clinical + Labs	351	24
Upper Middle	Cutland, 2015	South Africa	Clinical + Labs	389	66
Upper Middle	Dramowski, 2015	South Africa	Clinical + Labs	717	112
Upper Middle	Dramowski, 2015	South Africa	Labs	198	38
Upper Middle	Hentges, 2014	Brazil	Clinical + Labs	94	23
Upper Middle	Morkel, 2014	South Africa	Labs	54	25
Upper Middle	Ozkan, 2014	Turkey	Clinical + Labs	151	22
Upper Middle	Rugolo, 2014	Brazil	Clinical + Labs	702	213
Upper Middle	Akdag, 2014	Turkey	Clinical + Labs	51	2
Upper Middle	Thatrimontrichai, 2014	Thailand	Labs	241	43
Upper Middle	Turner, 2013	Thailand	Clinical	174	0
Upper Middle	Ballot, 2013	South Africa	Labs	59	27
Upper Middle	Al-Talib, 2013	Iraq	Clinical + Labs	398	36
Upper Middle	Ballot , 2012	South Africa	Clinical + Labs	181	40
Upper Middle	Freitas, 2012	Brazil	Clinical + Labs	76	15
Upper Middle	Leal, 2012	Mexico	Goldstein et al	514	49
Upper Middle	DPhil, 2012	South Africa	Clinical + Labs	323	18
Upper Middle	Afsharpaiman, 2012	Iran	Clinical + Labs	84	23
Upper Middle	Aletayeb, 2011	Iran	Clinical + Labs	153	82
Upper Middle	Kardana, 2011	Indonesia	Clinical + Labs	138	39
Upper Middle	Mutlu, 2011	Turkey	Clinical + Labs	116	23
Upper Middle	Yilmaz, 2010	Turkey	Clinical + Labs	374	59
Upper Middle	Tansug, 2010	Turkey	Labs	601	146
Upper Middle	Bas, 2010	Turkey	Clinical + Labs	100	38
Low Middle	Tank, 2019	Kenya	Clinical	320	80
Low Middle	Fahmey, 2019	Egypt	Clinical + Labs	50	19
Low Middle	Martin , 2019	India	Clinical + Labs	251	71
Low Middle	Charoo, 2019	India	Clinical + Labs	64	35
Low Middle	Arya, 2018	India	Clinical + Labs	298	57
Low Middle	Jajoo, 2018	India	Clinical + Labs	1416	153

Low Middle	Shimy, 2018	Egypt	Clinical + Labs	96	30
Low Middle	Kannan, 2018	India	Clinical + Labs	54	16
Low Middle	Fahmey, 2018	Egypt	Clinical + Labs	60	12
Low Middle	Bandyopadhyay, 2018	India	Labs	183	54
Low Middle	Singh, 2018	India	Labs	80	31
Low Middle	Ahmad, 2018	Pakistan	Clinical + Labs	151	10
Low Middle	Kumar, 2018	India	Clinical + Labs	297	87
Low Middle	Banupriya, 2018	India	Clinical + Labs	75	13
Low Middle	Khattab, 2018	Egypt	Clinical + Labs	60	19
Low Middle	Ellahony, 2017	Egypt	Clinical + Labs	500	190
Low Middle	Shabaan, 2017	Egypt	Clinical + Labs	51	16
Low Middle	Newton, 2017	India	Clinical + Labs	67	11
Low Middle	Shobowale, 2017	Nigeria	Clinical + Labs	100	12
Low Middle	Arowosegbe , 2017	Nigeria	Clinical + Labs	85	27
Low Middle	DPhil, 2017	Kenya	Clinical + Labs	995	150
Low Middle	Chohan, 2017	Pakistan	Clinical	63	9
Low Middle	Ahmad, 2017	Pakistan	Clinical + Labs	744	101
Low Middle	Jasani, 2016	India	Clinical + Labs	62	21
Low Middle	Investigators of the Delhi Neonatal Infection Study (DeNIS), 2016	India	Clinical + Labs	1934	496
Low Middle	Kabwe, 2016	Zambia	Clinical + Labs	313	134
Low Middle	Chandrasekaran, 2016	India	Clinical + Labs	59	10
Low Middle	Debbarma, 2016	India	Clinical	62	26
Low Middle	Pradhan, 2016	India	Clinical	63	13
Low Middle	Vishnu Bhat, 2016	India	Clinical + Labs	183	33
Low Middle	Ahmad, 2016	Pakistan	Clinical + Labs	374	47
Low Middle	Tran, 2015	Vietnam	Clinical + Labs	106	49
Low Middle	Aundhakar, 2015	India	Clinical + Labs	216	38
Low Middle	Mandavi, 2015	India	Clinical + Labs	100	25
Low Middle	El-Din, 2015	Egypt	Clinical + Labs	140	64
Low Middle	Dhanawade, 2015	India	Clinical + Labs	127	37
Low Middle	Shabaan, 2015	Egypt	Clinical + Labs	60	10
Low Middle	Gopichand, 2015	India	Clinical + Labs	62	19
Low Middle	Jajoo, 2015	India	Clinical + Labs	82	12
Low Middle	Datta, 2014	India	Clinical + Labs	105	22
Low Middle	Patel, 2014	India	Labs	226	34
Low Middle	Gill, 2014	Zambia	Clinical	208	20

Low Middle	Adly, 2014	Egypt	Clinical + Labs	112	27
Low Middle	Ramasamy, 2014	India	Clinical + Labs	90	13
Low Middle	Umate, 2014	India	Clinical + Labs	208	7
Low Middle	Catal, 2014	Turkey	Clinical + Labs	91	21
Low Middle	Ahmad, 2014	Pakistan	Clinical + Labs	469	68
Low Middle	Kumar, 2014	India	Labs	65	12
Low Middle	Umar, 2014	Pakistan	Clinical + Labs	250	89
Low Middle	Goheer, 2014	Pakistan	Clinical + Labs	82	6
Low Middle	Sheikh, 2014	Pakistan	Clinical	125	20
Low Middle	Rajendraprasad, 2013	India	Clinical + Labs	95	53
Low Middle	Shrima, 2013	Nepal	Clinical + Labs	192	71
Low Middle	Kaul, 2013	India	Clinical + Labs	102	20
Low Middle	S De, 2013	India	Labs	80	16
Low Middle	Kruse , 2013	Vietnam	Clinical + Labs	385	62
Low Middle	Thapa, 2013	Nepal	Clinical + Labs	186	15
Low Middle	Mehar, 2013	India	Clinical + Labs	63	18
Low Middle	Mehta, 2013	Nepal	Clinical + Labs	307	24
Low Middle	Iqbal, 2013	India	Clinical + Labs	194	51
Low Middle	Saleem, 2013	Pakistan	Labs	104	17
Low Middle	Stoesser, 2013	Cambodia	Clinical + Labs	65	24
Low Middle	Ahmad, 2012	Pakistan	Clinical + Labs	502	87
Low Middle	Basu, 2012	India	Clinical + Labs	55	14
Low Middle	Zaidi, 2012	Pakistan	Clinical	434	16
Low Middle	Bhat Y, 2012	India	Clinical + Labs	52	10
Low Middle	Viswanathan, 2012	India	Clinical + Labs	158	41
Low Middle	Abdeljelil, 2012	Tunisia	Clinical + Labs	127	80
Low Middle	Mhada, 2012	Tanzania	Clinical + Labs	74	18
Low Middle	Viswanathan, 2012	India	Clinical	216	29
Low Middle	Zakariya, 2012	India	Clinical + Labs	50	21
Low Middle	Shaw, 2012	Nepal	Clinical + Labs	100	4
Low Middle	Rao, 2012	India	Clinical	108	22
Low Middle	Gaurav, 2011	India	Clinical + Labs	102	17
Low Middle	Chiabi, 2011	Cameroon	Clinical + Labs	218	48
Low Middle	A.M., 2010	Pakistan	Clinical + Labs	60	24
Low Middle	Talbert, 2010	Kenya	Labs	505	167
Low Middle	Kayange, 2010	Tanzania	Clinical + Labs	300	57
Low Middle	Qureshi, 2010	Pakistan	Clinical + Labs	116	22
Low Middle	Mathur, 2010	India	Clinical	80	25
Low Middle	Tagare, 2010	India	Labs	115	16

Low Middle	Ogunlesi, 2010	Nigeria	Clinical + Labs	174	56
Low	Bunduki, 2019	Democratic Republic of the Congo	Goldstein et al	228	48
Low	Lenglet, 2018	Haiti	Clinical + Labs	257	191
Low	Tewabe, 2017	Ethiopia	Goldstein et al	225	9
Low	Buolos, 2017	Haiti	Clinical + Labs	708	163
Low	Molyneux, 2017	Malawi	Clinical	157	18
Low	Al-Shamahy, 2012	Yemen	Clinical + Labs	158	27
Multi	Santolaya, 2018	Multi	Clinical + Labs	89	36
Multi	Hibberd, 2016	Multi	Clinical	32088	4564
Multi	Fitzgerald, 2016	Multi	Goldstein et al	50	16
Multi	Hamer , 2015	Multi	Clinical + Labs	83	5
Multi	Rivera, 2015	Multi	Clinical + Labs	93	19
Multi	Fitzgerald, 2015	Multi	Goldstein et al	57	17
Multi	Mularoni, 2014	Multi	Clinical + Labs	391	134
Multi	Al-Taiar, 2013	Multi	Clinical + Labs	963	140
Multi	The INIS Collaborative Group, 2011	Multi	Clinical + Labs	1734	287

Supplementary Table 3. Risk of bias of randomized clinical trials

Income Group	Author and Publication Year	Domains						Overall Risk
		Sequence Generation	Allocation Concealment	Blinding of Participants and Personnel	Blinding of Outcome Assessor	Incomplete Outcome Data	Selective Outcome Reporting	
High	Fairchild, 2013	Low	Unclear	High	Low	Low	Low	**High**
High	Metsvaht, 2010	Low	Unclear	High	Low	Low	Low	**High**
Upper Middle	Akdag, 2014	Low	Low	Low	Low	Low	Low	**Low**
Upper Middle	DPhil, 2012	Low	Low	High	Low	Low	Low	**High**
Low Middle	Banupriya, 2018	Low	Low	Low	Low	Low	Low	**Low**
Low Middle	Shabaan, 2017	Low	Low	High	Low	Low	Low	**High**
Low Middle	Newton, 2017	Low	Low	High	Unclear	Low	Low	**High**
Low Middle	Vishnu Bhat, 2016	Low	Low	High	Low	Low	Low	**High**
Low Middle	Shabaan, 2015	Low	Low	Low	Low	Low	Low	**Low**
Low Middle	Gill, 2014	Unclear	Unclear	Unclear	Unclear	Low	Low	**Moderate**
Low Middle	Ramasamy, 2014	Low	Low	High	Low	Low	Low	**High**
Low Middle	Mehta, 2013	Low	Low	Low	Low	Low	Low	**Low**
Low Middle	Zaidi, 2012	Low	Low	High	Low	Low	Low	**High**
Low	Molyneux, 2017	Low	Low	High	Low	High	Low	**High**
Multi	The INIS Collaborative Group, 2011	Low	Low	Low	Low	Low	Low	**Low**

Supplementary Table 4. Risk of bias of observational studies

Income Group	Author and publication year	Domain			Overall risk
		Selection	Comparability	Outcome	
High	Abdellatif, 2019	Low	Low	Low	**Low**
High	Berardi, 2019	Low	Low	Low	**Low**
High	Zonnenberg, 2019	Low	Low	Low	**Low**
High	Al-Matary, 2019	Low	Low	Low	**Low**
High	Singh, 2019	Low	Low	Low	**Low**
High	Pruitt, 2019	Low	Low	Low	**Low**
High	Glikman, 2019	Low	Low	High	**Moderate**
High	Al Luhidan, 2019	Low	Low	Low	**Low**
High	Hamdy, 2019	Low	Low	Low	**Low**
High	Bohanon, 2018	Low	Low	Low	**Low**
High	Giannoni, 2018	Low	Low	Low	**Low**
High	Benedict, 2018	Low	Low	Low	**Low**
High	Glikman, 2018	Low	Low	Low	**Low**
High	Hsu, 2018	Low	Low	High	**Moderate**
High	Ting, 2018	Low	Low	Low	**Low**
High	Cantey, 2018	Low	Low	Low	**Low**
High	Al-Mouqdad, 2018	Low	Low	Low	**Low**
High	Kim, 2018	Low	Low	Low	**Low**
High	Carr, 2017	Low	Low	Low	**Low**
High	Agyeman, 2017	Low	Low	Low	**Low**
High	Kuzniewicz, 2017	Low	Low	Low	**Low**
High	Chen, 2017	Low	Low	Low	**Low**
High	Blanchard, 2017	Low	Low	Low	**Low**
High	Mendoza-Palomar, 2017	Low	Low	Low	**Low**
High	Wu, 2017	Low	Low	High	**Moderate**
High	Gowda, 2017	Low	Low	Low	**Low**
High	Hakansson, 2017	Low	Low	Low	**Low**
High	Ree, 2017	Low	Low	Low	**Low**
High	Drageset, 2017	Low	Low	Low	**Low**
High	Piening, 2017	Low	Low	Low	**Low**
High	Sanchez-Pinto, 2017	Low	Low	Low	**Low**
High	Fjalstad, 2016	Low	Low	Low	**Low**
High	DPhil, 2016	Low	Low	Low	**Low**
High	Ivady, 2016	Low	Low	Low	**Low**
High	Said, 2016	Low	Low	Low	**Low**
High	Bulkowstein, 2016	Low	Low	Low	**Low**
High	Pugni, 2016	Low	Low	Low	**Low**

High	Verstraete, 2016	Low	Low	Low	**Low**
High	Tsai, 2016	Low	Low	Low	**Low**
High	Lauterbach, 2016	Low	Low	Low	**Low**
High	Deshpande, 2016	Low	Low	Low	**Low**
High	Berardi, 2016	Low	Low	High	**Moderate**
High	Cobos-Carrascosa, 2015	Low	Low	Low	**Low**
High	Lee, 2015	Low	Low	Low	**Low**
High	Chmielarczyk, 2015	Low	Low	Low	**Low**
High	Bergin, 2015	Low	Low	Low	**Low**
High	Tsai, 2015	Low	Low	High	**Moderate**
High	Lai, 2015	Low	Low	High	**Moderate**
High	Yen, 2015	Low	Low	Low	**Low**
High	Hsu, 2015	Low	Low	High	**Moderate**
High	Schwab, 2015	Low	Low	Low	**Low**
High	Hsiu, 2015	Low	Low	High	**Moderate**
High	Mitsiakos, 2015	Low	Low	Low	**Low**
High	Shah, 2015	Low	Low	Low	**Low**
High	Schlapbach, 2015	Low	Low	Low	**Low**
High	Lutsar, 2014	Low	Low	Low	**Low**
High	Mitt, 2014	Low	Low	Low	**Low**
High	Oeser, 2014	Low	Low	Low	**Low**
High	Bamberger, 2014	Low	Low	Low	**Low**
High	Levit, 2014	Low	Low	High	**Moderate**
High	Chen, 2014	Low	Low	Low	**Low**
High	Bizzarro, 2014	Low	Low	Low	**Low**
High	Verstraete, 2014	Low	Low	Low	**Low**
High	Tsai, 2014	Low	Low	High	**Moderate**
High	Chu, 2014	Low	Low	Low	**Low**
High	Tsai, 2014	Low	Low	Low	**Low**
High	Natarajan, 2014	Low	Low	Low	**Low**
High	Balamuth, 2014	Low	Low	Low	**Low**
High	Dolapo, 2014	Low	Low	Low	**Low**
High	Adams-Chapman, 2013	Low	High	Low	**Moderate**
High	Ahmed, 2013	Low	Low	Low	**Low**
High	Wynn, 2013	Low	Low	Low	**Low**
High	Haan, 2013	Low	High	Low	**Moderate**
High	Ergaz, 2013	Low	Low	Low	**Low**
High	Hammoud, 2013	Low	Low	Low	**Low**
High	Luthander, 2013	Low	Low	Low	**Low**
High	Prieto, 2013	Low	Low	High	**Moderate**
High	Sood, 2012	Low	High	Low	**Moderate**

High	Hornik, 2012	Low	Low	Low	**Low**
High	Hammoud, 2012	Low	Low	Low	**Low**
High	Livorsi, 2012	Low	Low	Low	**Low**
High	Shane, 2012	Low	Low	Low	**Low**
High	Tsai, 2012	Low	Low	Low	**Low**
High	Morioka, 2012	Low	Low	Low	**Low**
High	Ahmed, 2012	Low	Low	Low	**Low**
High	Grisaru-Soen, 2012	Low	High	High	**Moderate**
High	Villa, 2012	Low	Low	Low	**Low**
High	Wong, 2012	Low	Low	Low	**Low**
High	Vergnano, 2011	Low	Low	Low	**Low**
High	Stoll, 2011	Low	Low	Low	**Low**
High	Al-Taiar, 2011	Low	Low	Low	**Low**
High	Weston, 2011	Low	Low	Low	**Low**
High	van der Broek, 2011	Low	Low	High	**Moderate**
High	Schlapbach, 2011	Low	High	Low	**Moderate**
High	Guilbert, 2010	Low	Low	Low	**Low**
High	Klinger, 2010	Low	Low	Low	**Low**
High	Auriti, 2010	Low	High	Low	**Moderate**
Upper Middle	Macooie, 2019	Low	Low	Low	**Low**
Upper Middle	Buyuktiryaki, 2019	Low	Low	High	**Moderate**
Upper Middle	Freitas, 2019	Low	Low	Low	**Low**
Upper Middle	Leal, 2019	Low	Low	Low	**Low**
Upper Middle	Varljen, 2019	Low	High	Low	**Moderate**
Upper Middle	Celik, 2019	Low	Low	Low	**Low**
Upper Middle	Stranieri, 2018	Low	Low	Low	**Low**
Upper Middle	Escalante, 2018	Low	Low	Low	**Low**
Upper Middle	Olugbuyi, 2018	Low	Low	Low	**Low**
Upper Middle	Yusef, 2018	Low	Low	High	**Moderate**
Upper Middle	Tarakci, 2018	Low	Low	Low	**Low**
Upper Middle	Thomas, 2018	Low	Low	High	**Moderate**
Upper Middle	Neira, 2018	Low	Low	Low	**Low**
Upper Middle	Softic, 2017	Low	Low	Low	**Low**
Upper Middle	Siavashi, 2017	Low	Low	High	**Moderate**
Upper Middle	Fu, 2017	Low	Low	Low	**Low**
Upper Middle	Cagan, 2017	Low	Low	Low	**Low**
Upper Middle	Zhou, 2016	Low	Low	High	**Moderate**
Upper Middle	Arizaga-Ballesteros, 2015	Low	Low	High	**Moderate**
Upper Middle	Mosayebi, 2015	Low	Low	Low	**Low**
Upper Middle	Cutland, 2015	Low	Low	Low	**Low**
Upper Middle	Turhan, 2015	Low	Low	Low	**Low**

Upper Middle	Dramowski, 2015	Low	Low	Low	**Low**
Upper Middle	Dramowski, 2015	Low	Low	Low	**Low**
Upper Middle	Hentges, 2014	Low	Low	Low	**Low**
Upper Middle	Ozkan, 2014	Low	Low	Low	**Low**
Upper Middle	Rugolo, 2014	Low	Low	Low	**Low**
Upper Middle	Morkel, 2014	Low	Low	Low	**Low**
Upper Middle	Thatrimontrichai, 2014	Low	Low	Low	**Low**
Upper Middle	Turner, 2013	Low	Low	Low	**Low**
Upper Middle	Ballot, 2013	Low	Low	Low	**Low**
Upper Middle	Al-Talib, 2013	Low	Low	Low	**Low**
Upper Middle	Freitas, 2012	Low	Low	Low	**Low**
Upper Middle	Ballot, 2012	Low	Low	High	**Moderate**
Upper Middle	Leal, 2012	Low	Low	Low	**Low**
Upper Middle	Afsharpaiman, 2012	Low	Low	Low	**Low**
Upper Middle	Aletayeb, 2011	Low	Low	Low	**Low**
Upper Middle	Kardana, 2011	Low	Low	Low	**Low**
Upper Middle	Mutlu, 2011	Low	Low	High	**Moderate**
Upper Middle	Bas, 2010	Low	Low	Low	**Low**
Upper Middle	Yilmaz, 2010	Low	Low	Low	**Low**
Upper Middle	Tansug, 2010	Low	Low	Low	**Low**
Lower Middle	Tank, 2019	High	Low	High	**Moderate**
Lower Middle	Fahmey, 2019	Low	High	Low	**Moderate**
Lower Middle	Martin, 2019	High	Low	Low	**Moderate**
Lower Middle	Charoo, 2019	Low	Low	Low	**Low**
Lower Middle	Kannan, 2018	Low	High	Low	**Moderate**
Lower Middle	Arya, 2018	Low	Low	Low	**Low**
Lower Middle	Jajoo, 2018	Low	Low	Low	**Low**
Lower Middle	Shimy, 2018	Low	Low	Low	**Low**
Lower Middle	Fahmey, 2018	Low	High	Low	**Moderate**
Lower Middle	Singh, 2018	Low	High	Low	**Moderate**
Lower Middle	Khattab, 2018	Low	Low	Low	**Low**
Lower Middle	Bandyopadhyay, 2018	Low	Low	Low	**Low**
Lower Middle	Ahmad, 2018	Low	Low	Low	**Low**
Lower Middle	Kumar, 2018	Low	Low	Low	**Low**
Lower Middle	Shobowale, 2017	Low	Low	Low	**Low**
Lower Middle	Arowosegbe, 2017	Low	Low	High	**Moderate**
Lower Middle	Chohan, 2017	Low	Low	Low	**Low**
Lower Middle	Ahmad, 2017	Low	Low	Low	**Low**
Lower Middle	Ellahony, 2017	Low	Low	Low	**Low**
Lower Middle	DPhil, 2017	Low	Low	Low	**Low**

Lower Middle	Kabwe, 2016	High	Low	Low	**Moderate**
Lower Middle	Debbarma, 2016	Low	Low	Low	**Low**
Lower Middle	Investigators of the Delhi Neonatal Infection Study (DeNIS), 2016	Low	Low	Low	**Low**
Lower Middle	Pradhan, 2016	Low	Low	Low	**Low**
Lower Middle	Jasani, 2016	Low	Low	Low	**Low**
Lower Middle	Chandrasekaran, 2016	Low	Low	Low	**Low**
Lower Middle	Ahmad, 2016	Low	Low	High	**Moderate**
Lower Middle	Tran, 2015	Low	Low	Low	**Low**
Lower Middle	Mandavi, 2015	Low	Low	Low	**Low**
Lower Middle	El-Din, 2015	Low	Low	Low	**Low**
Lower Middle	Gopichand, 2015	Low	Low	Low	**Low**
Lower Middle	Jajoo, 2015	Low	Low	Low	**Low**
Lower Middle	Aundhakar, 2015	Low	Low	High	**Moderate**
Lower Middle	Dhanawade, 2015	Low	Low	Low	**Low**
Lower Middle	Sheikh, 2014	Low	Low	High	**Moderate**
Lower Middle	Datta, 2014	Low	Low	Low	**Low**
Lower Middle	Adly, 2014	Low	High	Low	**Moderate**
Lower Middle	Umate, 2014	Low	Low	Low	**Low**
Lower Middle	Patel, 2014	Low	Low	Low	**Low**
Lower Middle	Catal, 2014	High	Low	Low	**Moderate**
Lower Middle	Ahmad, 2014	Low	Low	Low	**Low**
Lower Middle	Kumar, 2014	Low	Low	Low	**Low**
Lower Middle	Umar, 2014	Low	Low	Low	**Low**
Lower Middle	Goheer, 2014	Low	Low	Low	**Low**
Lower Middle	Thapa, 2013	High	Low	Low	**Moderate**
Lower Middle	Shrima, 2013	Low	Low	Low	**Low**
Lower Middle	Kruse, 2013	Low	Low	Low	**Low**
Lower Middle	Rajendraprasad, 2013	Low	Low	Low	**Low**
Lower Middle	Kaul, 2013	Low	Low	Low	**Low**
Lower Middle	S De, 2013	Low	Low	Low	**Low**
Lower Middle	Mehar, 2013	Low	Low	High	**Moderate**
Lower Middle	Iqbal, 2013	Low	Low	Low	**Low**
Lower Middle	Saleem, 2013	Low	Low	Low	**Low**
Lower Middle	Stoesser, 2013	Low	Low	Low	**Low**
Lower Middle	Mhada, 2012	Low	Low	Low	**Low**
Lower Middle	Basu, 2012	Low	High	Low	**Moderate**
Lower Middle	Bhat Y, 2012	Low	Low	Low	**Low**
Lower Middle	Viswanathan, 2012	Low	Low	Low	**Low**

Lower Middle	Viswanathan, 2012	Low	Low	Low	**Low**
Lower Middle	Zakariya, 2012	Low	Low	Low	**Low**
Lower Middle	Rao, 2012	Low	Low	Low	**Low**
Lower Middle	Ahmad, 2012	Low	Low	Low	**Low**
Lower Middle	Abdeljelil, 2012	Low	Low	Low	**Low**
Lower Middle	Shaw, 2012	Low	Low	Low	**Low**
Lower Middle	Gaurav, 2011	Low	Low	Low	**Low**
Lower Middle	Chiabi, 2011	Low	Low	Low	**Low**
Lower Middle	Kayange, 2010	Low	Low	Low	**Low**
Lower Middle	Qureshi, 2010	Low	Low	Low	**Low**
Lower Middle	A.M., 2010	Low	Low	Low	**Low**
Lower Middle	Talbert, 2010	Low	Low	Low	**Low**
Lower Middle	Mathur, 2010	Low	Low	Low	**Low**
Lower Middle	Tagare, 2010	Low	Low	Low	**Low**
Lower Middle	Ogunlesi, 2010	Low	Low	Low	**Low**
Low	Bunduki, 2019	Low	Low	Low	**Low**
Low	Lenglet, 2018	High	Low	Low	**Moderate**
Low	Tewabe, 2017	Low	Low	High	**Moderate**
Low	Buolos, 2017	High	Low	Low	**Moderate**
Low	Al-Shamahy, 2012	Low	Low	Low	**Low**
Multi	Santolaya, 2018	Low	Low	Low	**Low**
Multi	Fitzgerald, 2016	Low	Low	Low	**Low**
Multi	Hibberd, 2016	Low	Low	Low	**Low**
Multi	Weiss, 2015	Low	Low	Low	**Low**
Multi	Rivera, 2015	Low	Low	Low	**Low**
Multi	Hamer, 2015	Low	Low	Low	**Low**
Multi	Mularoni, 2014	Low	Low	Low	**Low**
Multi	Al-Taiar, 2013	Low	Low	Low	**Low**
